# Advancing tuberculosis management: the role of predictive, preventive, and personalized medicine

**DOI:** 10.3389/fmicb.2023.1225438

**Published:** 2023-10-04

**Authors:** Matúš Dohál, Igor Porvazník, Ivan Solovič, Juraj Mokrý

**Affiliations:** ^1^Biomedical Centre Martin, Jessenius Faculty of Medicine in Martin, Comenius University in Bratislava, Martin, Slovakia; ^2^National Institute of Tuberculosis, Lung Diseases and Thoracic Surgery, Vyšné Hágy, Slovakia; ^3^Faculty of Health, Catholic University in Ružomberok, Ružomberok, Slovakia; ^4^Department of Pharmacology, Jessenius Faculty of Medicine in Martin, Comenius University in Bratislava, Martin, Slovakia

**Keywords:** tuberculosis, PPPM, artificial intelligence, whole genome sequencing, treatment failure

## Abstract

Tuberculosis is a major global health issue, with approximately 10 million people falling ill and 1.4 million dying yearly. One of the most significant challenges to public health is the emergence of drug-resistant tuberculosis. For the last half-century, treating tuberculosis has adhered to a uniform management strategy in most patients. However, treatment ineffectiveness in some individuals with pulmonary tuberculosis presents a major challenge to the global tuberculosis control initiative. Unfavorable outcomes of tuberculosis treatment (including mortality, treatment failure, loss of follow-up, and unevaluated cases) may result in increased transmission of tuberculosis and the emergence of drug-resistant strains. Treatment failure may occur due to drug-resistant strains, non-adherence to medication, inadequate absorption of drugs, or low-quality healthcare. Identifying the underlying cause and adjusting the treatment accordingly to address treatment failure is important. This is where approaches such as artificial intelligence, genetic screening, and whole genome sequencing can play a critical role. In this review, we suggest a set of particular clinical applications of these approaches, which might have the potential to influence decisions regarding the clinical management of tuberculosis patients.

## 1. Introduction

Tuberculosis (TB) is an infectious disease caused by the bacterium *Mycobacterium tuberculosis* (*Mtb*). The World Health Organization (WHO) formulated the End TB Strategy to achieve the ultimate eradication of TB. The strategy gained approval in 2014 from the 67^th^ World Health Assembly and aims to terminate the global TB epidemic by 2035 (WHO, 2021). Initially, the strategy has aimed to diminish the number of individuals afflicted with TB by 90%, along with lowering the mortality rate by 95% and safeguarding families from the adverse outcomes of TB. Predictive, preventive, and personalized medicine (PPPM) can significantly contribute to achieving this goal (Sadkovsky et al., 2014; Khan and Das, 2022). This approach emphasizes the use of advanced technologies and data analysis to predict an individual’s susceptibility to a disease, prevent its onset, and personalize treatment to optimize expected outcomes (Huang et al., 2022). In the case of TB, PPPM plays an important role in several ways:

1. Predictive medicine in the context of TB refers to the application of data analysis and advanced screening techniques to identify individuals with a high probability of contracting TB. This strategy can also predict the risk of treatment failure and improve TB management strategies. Predictive models are developed using various data sources, such as clinical, genetic, and environmental data (MacNeil et al., 2019; Martinez et al., 2020).
2. Preventive medicine involves strategies to prevent the onset of a disease. Various prophylactic measures can be implemented in the management of TB and prevention of the development of active TB. The most common is the use of the Bacillus Calmette-Guérin (BCG) vaccine. Other preventive measures include identifying and treating latent TB infection (LTBI) in individuals who have been exposed to *Mtb* but have not yet developed active TB (Pooransingh and Sakhamuri, 2020; Berrocal-Almanza et al., 2022; Migliori et al., 2022).
3. Personalized (precision) medicine refers to the approach of tailoring medical treatment to individual patients by considering their unique traits and requirements. In TB, personalized medicine is mostly used to optimize treatment regimens for patients based on *Mtb* resistance and individual genetic variations in TB patients in responding to drugs (such as drug metabolism efficacy; Joshi, 2011). Many studies have shown an association between the genotype of *Mtb* and a higher risk of developing resistance. For example, the Beijing lineage is currently considered the most prevalent among multidrug-resistant (MDR) strains (Zhou et al., 2017; Karmakar et al., 2019; Bakuła et al., 2023). Also, *Mtb* strains of this lineage are predominantly linked to active TB and carry an elevated risk of treatment failure (Keikha and Majidzadeh, 2021). Genetic testing can identify patients with a higher risk of acquiring drug-resistant TB or experiencing adverse effects, and the treatment regimen can be customized accordingly (Richardson et al., 2018). The treatment regimens can be tailored based on a patient’s clinical characteristics, such as age, alcoholism, anaemia, and HIV co-infection, as well as sodium, iron, and albumin deficiency (Resende and dos Santos-Neto, 2015). In addition, measurement of plasma concentrations of anti-tuberculosis can be implemented in adjusting the doses of respective drugs in case of various interactions or individual discrepancies despite using their recommended doses (Pršo et al., 2023).

This literature review includes the findings of the latest studies aimed at PPPM strategies, including artificial intelligence (AI), genetic screening, microRNA (miRNA) and whole genome sequencing (WGS). Importantly, we explore the possibility of applying these approaches in enhancing TB diagnosis, treatment, and prevention by identifying individuals at high risk, preventing the spread of the disease, and personalizing treatment regimens to individual patients.

## 2. Transforming tuberculosis care with artificial intelligence-powered PPPM

Identifying and treating individuals at high risk of TB infection or disease progression are currently considered the most cost-effective measures for TB control and prevention (Kielmann et al., 2020). Among the tools available for these purposes, the latest analytical tools are currently demonstrating the greatest efficacy. Out of all the available analytical instruments, artificial intelligence (AI) is considered the most potent and encouraging for humanity. AI employs mathematical techniques such as ‘machine learning’ to learn patterns in training data and then applies this knowledge to make decisions when similar patterns are detected in new data (Silver et al., 2017; Fitzpatrick et al., 2020). Simultaneous advancements in information technology (IT) infrastructure and the processing power of mobile computing have created optimism that AI could offer possibilities to tackle health issues also in low- and middle-income countries (LMICs; Wahl et al., 2018).

In TB screening, chest radiography is recommended and the most preferred method globally (WHO, 2021; Moodley et al., 2022). Despite its usefulness, the main constraint of using chest X-ray (CXR) for screening TB patients in low-resource, high-burden areas is the shortage of radiologists, which has led to its limited implementation (Pande et al., 2015). In 2021, the WHO revised its TB screening guidelines, suggesting computer-aided detection software to evaluate digital CXR for predicting the likelihood of TB-related signs. This leads to better diagnostic decision-making, screening, and triaging TB in individuals aged 15 years and above (WHO, 2022). Over the past decade, AI-assisted diagnostic systems have progressed and advanced rapidly. Various medical-image-analyzing AI algorithms based on deep learning and deep convolutional neural networks (DCNNs), have been utilized for interpreting radiographs (Lakhani and Sundaram, 2017). A recent study highlighted the potential of a deep learning web-based diagnostic assistant in the prediction of TB in HIV-positive patients without the need for advanced radiological expertise (Rajpurkar et al., 2020). Acharya and colleagues created a normalization-free deep learning network model that enables the diagnosis and classification of TB with a sensitivity and specificity of 91.81 and 98.42%, respectively, on a dataset containing multiple classes. In addition, the model achieves an accuracy of 96% for binary classification (Acharya et al., 2022). The most extensive study using five commercial AI algorithms (AD4TB, InferRead DR, Lunit INSIGHT CXR, JF CXR-1, qXR) was performed in Bangladesh. Furthermore, aside from the fact that all the algorithms demonstrated a sensitivity of over 90%, the findings of the investigation revealed that utilizing these tools can potentially diminish the need for costly molecular diagnostic tests (e.g., Xpert MTB/RIF, Cepheid, United States) by up to 50% (Qin et al., 2021). The DCNN algorithm ResNet exhibited exceptional performance in the timely detection of active TB, a critical factor in managing the alarming increase in TB incidence (Nijiati et al., 2022). The deep learning method was utilized to distinguish between TB, COVID-19, and lung adenocarcinoma in patients with abnormal CXRs. The findings demonstrated a significant level of sensitivity and highlighted the potential utilization of AI methodologies to diagnose emerging respiratory infections (Feng et al., 2021; Yoo et al., 2021). Numerous research studies have been conducted to create AI predictive models that can differentiate between susceptible TB and multidrug-resistant TB using CXRs. The results indicate variable performance, with the area under the curve (AUC) values ranging from 0.74 to 0.85 (Jaeger et al., 2018; Karki et al., 2021). In addition, portable X-rays (MINE 2 HDT, Gwangju, Republic of Korea; Xair FDR XD2000, Fujifilm Corporation, Tokyo, Japan; Delft Ultra, Delft Imaging Systems, Netherlands) are currently available on the market, which have been confirmed to be useful in the search for active cases of TB in high-burden and rural areas (Vo et al., 2021; Odume et al., 2022). The Delft Ultra and Xair systems can integrate with software platforms that support AI-driven interpretation. Hence, the utilization of this tool can effectively contribute to the early detection of TB and facilitate the swift initiation of treatment. The potential hazard for medical personnel lies in their exposure to radiation, albeit in the case of portable X-rays, the risk is significantly diminished compared to that posed by a traditional apparatus (Kamal et al., 2023).

Coughing is another common symptom of pulmonary TB (Farina et al., 2022). AI algorithms can undergo training to analyze audio recordings of cough sounds and recognize patterns that are suggestive of TB infection. This method, referred to as “acoustic cough analysis,” possesses immense potential as a non-invasive and cost-efficient technique for TB screening. The accuracy of cough monitoring achieved high accuracy, however, and AI methods for diagnosing TB depend on various factors such as the quality and diversity of the training data, the specific AI algorithms used, and the stage of development and validation of the methods (Botha et al., 2018; Pahar et al., 2021; Zimmer et al., 2022). Additionally, AI can assist in epidemiological monitoring by examining cough data obtained from diverse sources, including wearable devices or mobile applications. Through the analysis of cough patterns in particular regions or communities, health authorities can obtain valuable information regarding the prevalence of TB, identify areas at high risk, and allocate resources more efficiently (Huddart et al., 2023). Despite its potential, the utilization of acoustic cough analysis and artificial intelligence (AI) in diagnosing and managing TB is currently in the research and development stage. Continuous studies and collaborations involving medicine, machine learning, and public health experts are essential to enhance and validate these methodologies. Addressing challenges such as personal data, standardization of cough recording protocols, and equitable access to AI technologies are crucial for their widespread implementation.

AI can predict the onset of TB and assess the efficacy of treatment by analyzing patient data such as demographics, medical history, and biomarkers. Asad et al. (2020) employed a machine learning model to predict the likelihood of treatment failure by analyzing various factors, such as social and health-related attributes. Similarly, Samson Balogun et al. (2021) tested 5 different machine learning models that performed well in classifying the TB treatment outcome (ranging between 67.5 and 73.4%). The latest research by Liao and colleagues has emphasized the potential of AI in anticipating side effects associated with the treatment of TB. The findings show that AI can identify acute hepatitis at an early stage in TB patients (based on levels of serum alanine aminotransferase, aspartate aminotransferase and total bilirubin), and also predict acute respiratory failure or death and may assist in clinical decision-making before these adverse effects occur (Liao et al., 2023). Moreover, Larkins-Ford et al. developed a mathematical model including a series of criteria to determine what drug combinations must be met for effective treatments when administered as three- or four-drug cocktails. This method can be used in the development of novel regimens, including twelve commonly used anti-TB drugs, to narrow down the potential combinations for subsequent pharmacokinetic/pharmacodynamic and preclinical studies (Larkins-Ford et al., 2022). To enhance medication adherence monitoring in TB patients, Sekandi et al. developed an AI model using video images of TB medication intake from Uganda and the rest of Africa. Their results can significantly contribute to determining whether the individuals have taken the pill or not, particularly in developing countries (Sekandi et al., 2023). It’s important to note that while AI can be a valuable tool in predicting TB onset and assessing treatment efficacy, it should always be used in conjunction with clinical expertise and human decision-making. AI models should be continuously validated and updated with new data to ensure their accuracy and reliability in real-world scenarios.

AI can also play an important role in TB prevention by helping to identify and track TB cases and predict outbreaks. Mandal et al. used AI algorithms to predict TB risk among TB patients’ household contacts in India. They found that the algorithm was able to accurately predict the risk of TB based on demographic and clinical data, including age, sex, body mass index, and history of exposure to TB (Mandal et al., 2020). During the COVID-19 pandemic, there was a significant increase in the development and deployment of AI models for digital contact tracing (Haneya et al., 2021; Almotairi et al., 2023). These models were used to track the spread of the virus and identify individuals who may have been exposed to the virus, allowing faster and more effective tracing. The success of these models has highlighted the potential of AI for tracking other infectious diseases, including TB (Shahroz et al., 2021).

**Figure 1:**
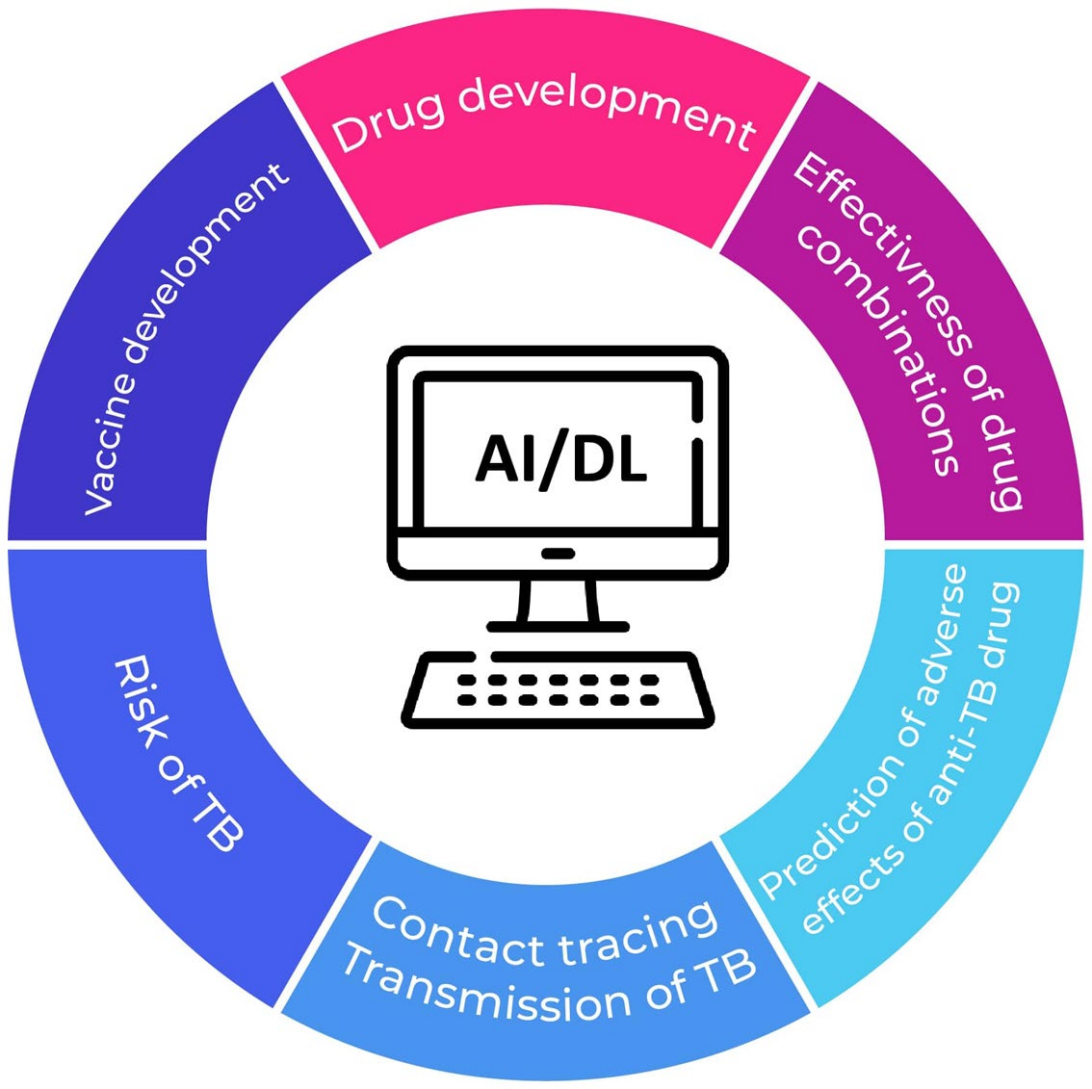
Potential applications of AI in preventing TB and mitigating the risk of treatment failure.

In summary, AI-driven methodologies, encompassing deep learning and other conventional machine learning techniques employed in the context of TB, offer a self-directed, convenient, and time-efficient approach to enhance diagnostic efficacy and precision, surpassing the performance of radiologists and other medical personnel (Figure 1). Nevertheless, the clinical applicability of these approaches requires further clarification, while challenges such as model reproducibility and data standardization need to be effectively tackled.

## 3. Utility of genetic screening and miRNA in PPPM for TB

### 3.1. Detecting particular mutations and miRNAs to predict and prevent active TB

Genetic screening can play a significant role in the management of TB. This approach can help identify individuals who are at higher risk of developing TB and personalize treatment regimens for individuals who have already been diagnosed with TB (Yan et al., 2022). The susceptibility of the host to TB has been linked to numerous genetic polymorphisms (Aravindan, 2019). Despite prior research linking several genetic polymorphisms to TB susceptibility, recent studies have identified numerous gene variations and microRNA (miRNA) biomarkers strongly associated with the risk of TB as well as the efficacy of treatment.

To enable more precise intervention in TB, it is crucial to identify biomarkers and genetic variants that can accurately predict the risk of developing active disease from latent TB infection (LTBI), as well as the progression of the infection. It is well-established that genetic factors in the host play a crucial role in the development of active TB. The majority of studies investigating the genes associated with immunity, including DC-SIGN, TLR1/2, vitamin D receptor, tumour necrosis factor, interleukin 1β, interferon γ, and HLA II molecules (Azad et al., 2012; Tervi et al., 2023). Moreover, Zhang et al. investigated the association between individual single nucleotide polymorphisms (SNPs) located within the rs1135216 and rs1057141 in the transporter-associated antigen processing gene (*TAP*)*1*, as well as rs2228396 in *TAP2*, and the likelihood of developing of pulmonary TB. According to their findings, rs1057141 may serve as a genetic indicator of decreased risk for TB in individuals aged 60 or older, whereas rs1135216 may be a potential genetic indicator for those under the age of 60 (Zhang et al., 2015). Xing et al. (2021) found a relationship between polymorphisms in cytochrome P450 (CYP450) and TB susceptibility. *CYP2C8* and *CYP2E1* variants were linked to a higher susceptibility to TB, implying the identification of these variants could be critical in defining new therapeutic strategies for chemoprevention. Recently, genetic variants in the cytokine genes (*IFGN*, *IL-12*, *IL14*, *TNFB*, and *IL1RA*) and transporter associated with *TAP* were associated with the susceptibility to pulmonary TB and genetic variants in *LIA4H*, *P2RX7*, *DCSIGN*, and *SP110* associated with susceptibility to LTBI (Abhimanyu et al., 2023; Lu et al., 2023). While these studies make valuable contributions to expanding the understanding of the genetic basis of PTB and EPTB manifestations, further research is warranted with larger sample sizes and diverse populations. Moreover, the identification of a whole-blood-based host genetic signature comprising four transcripts that predict progression to TB is promising and represents a big step forward in the personalization of TB treatment. This simple PCR test may also help predict TB transmission (Suliman et al., 2018).

The results of Xin et al. (2022) showed a possible correlation in the prediction of the development of active TB from LTBI with circulating miRNA hsa-miR-451a levels. The function of certain additional miRNAs (e.g., 146a, 149) in the risk of active TB progression has been elucidated; however, these studies were conducted with restricted sample sizes (Li et al., 2011; Zhang et al., 2015; Sinigaglia et al., 2020). Similarly, (Angria et al., 2022) discovered that assessing the expression of miRNA-29a-3p could serve as a screening method for individuals with LTBI. A recent study revealed the potential of miRNAs in predicting extrapulmonary forms of TB. The hsa-mir-425-5p miRNA expression levels in patients with lymph node TB were significantly higher compared to the other groups (including patients with LTBI and pulmonary TB; Massi et al., 2023). Research focusing on specific miRNA profiles for distinguishing latent LTBI, extrapulmonary- and pulmonary TB remains relatively limited but holds significant importance. This is because diagnosing extrapulmonary TB can be challenging in clinical settings, as conventional methods like AFB smear and culture are not always effective. Moreover, paucibacillary samples such as cerebrospinal fluid and aspirates are commonly encountered, contributing to milder forms of infection. Further exploration of miRNA in this context is expected to yield substantial benefits, particularly in the development of miRNA-based vaccines, biomarkers, and host-directed therapeutic approaches.

In the PPPM context, preventing excessive inflammation and death in TB patients is necessary. miRNA-27b-3p, miRNA-223-3p, and miR-99b-5p may play an important role in achieving these goals (Sinigaglia et al., 2020). By inhibiting the production of pro-inflammatory agents and nuclear factor kappa B activity, miR-27b-3p helps to decrease bacterial load and prevent excessive inflammation during *Mtb* infection (Liang et al., 2018). Lower miR-99b-5p expression results in decreased bacterial proliferation in dendritic cells and the enhancement of several pro-inflammatory cytokines, including IL-6, IL-12, and IL-1β (Singh and Goyal, 2013). We believe that identifying relevant miRNAs whose expression consistently correlates with the onset of active TB or divergent response to treatments could hold considerable clinical significance. Their collective efficacy lies in establishing routine diagnostic screening tests that exhibit substantial predictive capability, thereby enhancing the accuracy of existing tests predominantly reliant on the tuberculin skin test or interferon-gamma release assay (IGRA) which do not have a high accuracy for predicting active TB based on WHO recommendations (Gualano et al., 2019).

Integrating genetic screening and miRNA analysis can provide a more comprehensive understanding of TB pathogenesis and individualized patient management. By identifying genetic variants associated with TB susceptibility and miRNAs related to disease progression or treatment response, researchers can develop predictive models to guide personalized treatment decisions. This approach may also help identify novel therapeutic targets for drug development.

### 3.2. Advantages of genetic analysis in the individualized treatment of TB

In personalized medicine, pharmacogenetics and pharmacogenomics are two emerging fields that play a critical role in predicting individual responses to medication. Research has shown that differences in pharmacokinetic (PK) vulnerability to drugs among individuals contributed to some unfavorable outcomes, even in patients who followed the prescribed dosage regimen. This finding challenges the traditional idea that treatment failure, relapse, and the development of antimicrobial resistance are mostly attributed to non-adherence, thereby highlighting the need for genetic screening in TB patients (Sloan et al., 2017; Khan et al., 2022).

Several studies have also indicated a link between different genetic mutations and alterations in the plasma concentrations and adverse effects of first- and second-line anti-tuberculosis drugs in TB patients. Adverse reactions to the anti-tuberculosis drugs frequently include hepatotoxicity, severe cutaneous reactions (e.g., Stevens-Johnson syndrome, toxic epidermal necrolysis, acute generalized exanthematous pustulosis, maculopapular exanthema), queasiness, vomiting, purpura, lethargy, dizziness, abdominal discomfort, rare cases of osteomalacia, hyperuricaemia, rare incidents of acute kidney failure, rare instances of anemia, gastrointestinal or neurological disorders (Gholami et al., 2006; Tostmann et al., 2008; World Health Organization, 2010; Yu et al., 2017; Minardi et al., 2021). Hepatotoxicity is the most critical (Huai et al., 2019). Genetic factors have been recently widely studied to predict the risk of developing a drug-induced liver injury. At present, liver toxicity has been predominantly linked with variations in drug metabolism genes such as N-Acetyltransferase 2 (*NAT2*), *CYP2E1*, pregnane X receptor (*PXR*), and glutathione S-transferase (*GST*; Roy et al., 2001; Leiro et al., 2008). A better understanding of these mutations could facilitate in designing and developing a more effective personalized treatment for TB (Meitei et al., 2022). Lyu et al. (2019) described a significant correlation between single nucleotide polymorphisms (SNPs) in calcium signaling-related genes, specifically bradykinin receptor B2 (*BDKRB2*) and transforming growth factor beta 2 (*TGFB2*), and the onset of liver injury induced by anti-tuberculosis drugs. Moreover, performing genotyping on the *ABCB11* gene, which encodes the bile salt export pump (BSEP), could offer advantages for personalizing anti-tuberculosis treatment regimens (Cavaco et al., 2022). Regarding rifampicin, alterations in the solute carrier organic anion transporter family member 1B1 gene (*SLCO1B1*) have been extensively studied (Khan et al., 2022). Previous research showed that a genetic variant known as rs4149056 might decrease the expression of *SLCO1B1*, resulting in reduced uptake/transport activity of organic anion-transporting polypeptide 1B1 (*OATP1B1*) and higher levels of rifampicin in the bloodstream. Genetic screening of this variant may help to predict the increased rifampicin concentration (Niemi et al., 2011; Allegra et al., 2017). In contrast, patients carrying the rs11045819 or rs2306283 variant in S*LCO1B1* reached notably lower plasma levels of rifampicin compared to those with the wild-type genotype (Weiner et al., 2010; Dompreh et al., 2018). Similarly, Weiner et al. examined the impact of the –11187G > A mutation in the *SLCO1B1* gene on the pharmacokinetics of the second-line anti-tuberculosis drug moxifloxacin. The authors observed that patients carrying the variant exhibited significantly elevated Cmax values. This increase in the drug’s plasmatic concentration may contribute to the adverse effects of moxifloxacin, especially the prolongation of QT interval (Weiner et al., 2018). Song et al. (2013) found the c.-22263A > G mutation in the carboxylesterase (*CES2*) gene and described its correlation with elevated concentrations of rifampicin in the plasma of TB patients. Concerning isoniazid, the first step in the metabolism of this drug involves the non-inducible hepatic and intestinal enzyme NAT type 2, which is encoded by a highly polymorphic gene called the *NAT2* gene (Khan et al., 2013). Previous studies on genotyping the *NAT2* as a pharmacogenetic biomarker for the personalization of isoniazid therapeutic dosage revealed a direct correlation between the plasmatic concentration and the *NAT2* allele (Kinzig-Schippers et al., 2005; Fukino et al., 2008). In addition, the gene polymorphisms in *NAT2* have consistently demonstrated an association with an elevated risk of isoniazid-induced hepatotoxicity in various studies (Jaramillo-Valverde et al., 2022; Masiphephethu et al., 2022; Mohamed Noor et al., 2022). On the contrary, the study conducted by Kim et al. yielded results indicating that severe cutaneous adverse reactions associated with first-line anti-tuberculosis drugs are not linked to polymorphisms in *NAT2* or *CYP2E1* genes. However, these reactions are indeed associated with mutations in the *CYP2C9* and *CYP2C19* genes (Kim et al., 2011).

Among patients receiving drug-resistant TB therapy that includes aminoglycoside antibiotics, the most severe potential adverse effect is ototoxicity (Selimoglu, 2007). Previous studies have indicated that variations in mitochondrial DNA, particularly in the 12S rRNA genes, may be linked to increased susceptibility and toxicity to these antibiotics (Stocco et al., 2020). The m1555A > G and m.1494C > T variants in the 12 s rRNA gene have been extensively investigated and were conclusively associated with an increased risk of developing hearing loss after exposure to aminoglycosides (Guan, 2011; Zhang et al., 2013). It is hypothesized that several additional mitochondrial variations may eventually be identified as key contributors to the development of hearing loss. However, the complete biochemical mechanisms underlying this phenomenon have yet to be fully understood. These findings suggest that personalized antibiotic prescribing based on the patient’s 12 s rRNA genotype has the potential to lower the incidence of aminoglycoside-induced hearing loss in patients with drug-resistant TB (McDermott et al., 2022).

We recommend screening the established and well-defined genetic polymorphisms in the *CYP2E1* and *NAT2* genes, as their effects have been confirmed through robust association studies involving large population cohorts. As demonstrated in this review, ongoing research is investigating the association between polymorphisms in numerous candidate genes and adverse effects of anti-TB drugs. However, it is important to note that these associations are supported by limited studies with smaller sample sizes, often conducted in highly specific patient populations. Implementing genotyping tests as a part of a personalized medicine approach for TB treatment in high-endemic regions could be a crucial step toward achieving the “End-TB” goal by 2025 (Khan and Das, 2022). However, it’s important to emphasize that the inclusion of genetic screening for these factors may depend on several factors, including the availability of tests, cost-effectiveness, and the specific adverse effects of concern in the local population. Additionally, individual patient characteristics, such as liver function and comorbidities, should also be taken into consideration when assessing the risk of adverse effects and determining personalized treatment plans. Further investigation and consultation with experts in pharmacogenetics and personalized medicine are recommended to obtain the most relevant and up-to-date information for a particular clinical context (Sharma et al., 2022).

## 4. Unlocking the potential of next-generation sequencing in the context of PPPM in TB

Next,-generation sequencing (NGS) has emerged as a powerful tool for understanding the genetic background of various infectious diseases, including TB (Gordon et al., 2021). In the context of PPPM, NGS can transform and accelerate the delivery of personalized treatment to patients affected by TB, thus revolutionizing the way TB is diagnosed and treated.

**Figure 2:**
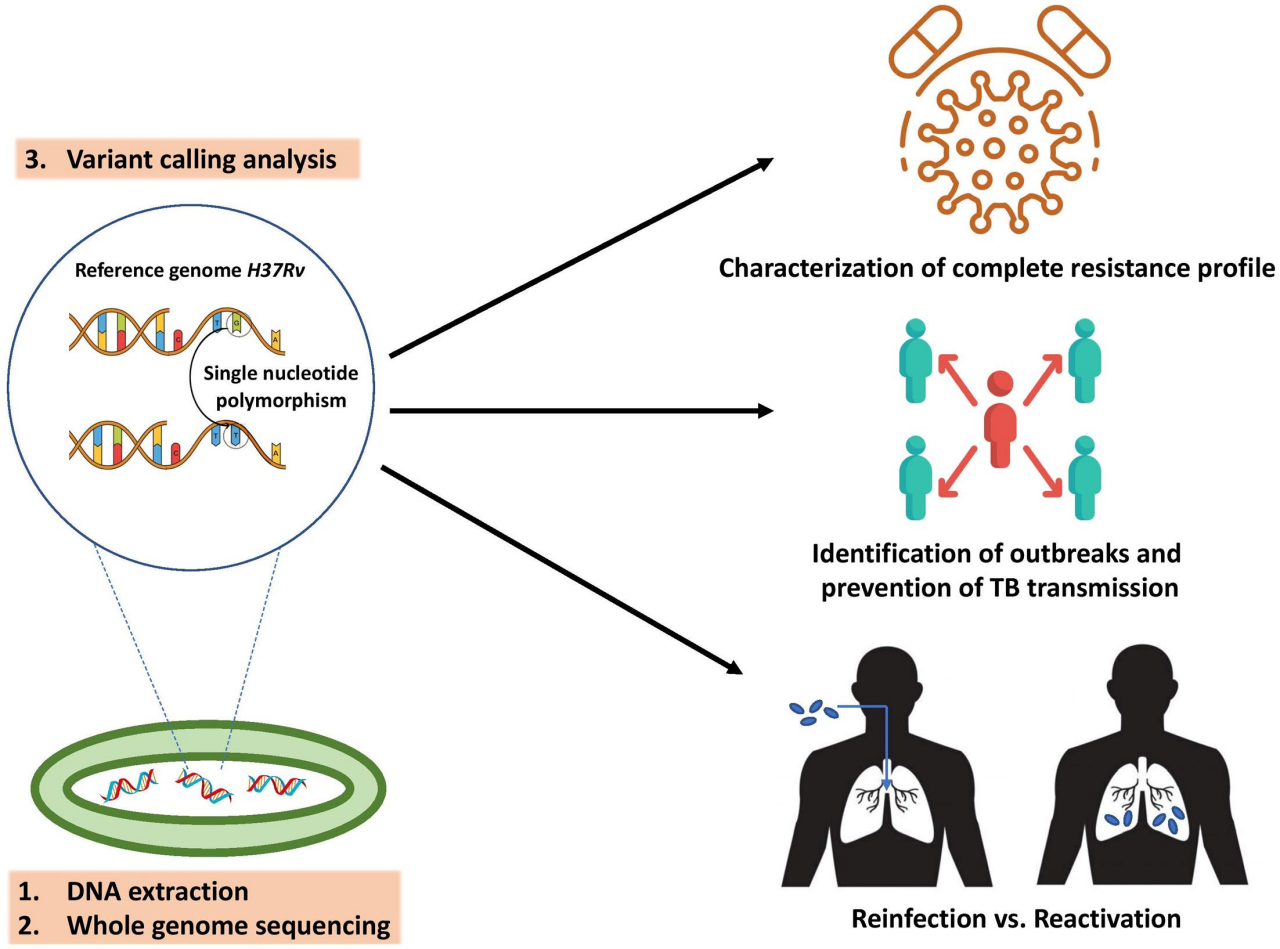
Key applications of *M. tuberculosis* WGS in PPPM include characterization of complete resistance profile to reach the highest treatment efficacy, determination of transmission chains and outbreaks to prevent the spread of TB, and distinguishing the cause of TB recurrence to guide TB control and treatment.

A more comprehensive drug susceptibility profile is needed to design an effective treatment plan for patients with drug-resistant TB (WHO, 2022). WGS has been identified as a highly promising tool for this particular purpose (Figure 2; Gröschel et al., 2018; Wu et al., 2020; Dohál et al., 2022). Cox et al. recently employed WGS to assess the accuracy of treatment regimens (derived from standard drug susceptibility testing and clinical information) in individuals with drug-resistant TB. Overall, 305 (24%) patients had MDR/rifampicin resistant (RR)-TB with second-line TB drug resistance, where the availability of WGS-derived drug susceptibility testing (DST) would have enabled more effective treatment personalization for these patients, such as reducing drug dosages or removing ineffective drugs (Cox et al., 2022; Xiao et al., 2023). Similarly, the results of Korhonen demonstrated that WGS could aid in the selection of optimal treatment regimens in the future, primarily for patients with resistance to ethambutol (Korhonen et al., 2022). The application of WGS in routine clinical practice also enables rapid identification of isoniazid monoresistance, reducing delays in treatment decisions and initiating WHO-recommended treatment for isoniazid-resistant TB (Park et al., 2022). In settings with a low incidence of TB, WGS reduced the time required for appropriate treatment modification, thus decreasing the expenses associated with hospitalization and treatment (Mugwagwa et al., 2021).

Bedaquiline and pretomanid, novel oral anti-tuberculosis drugs, have demosntrated excellent efficacy against both drug-susceptible and drug-resistant strains of *Mtb* and have been recommended by WHO (2022) as “reserved drug” for the treatment of MDR. As primary resistance to bedaquiline has been reported for several years, determining the sensitivity is essential for every patient being considered for a treatment regimen that includes this drug (Villellas et al., 2017). According to Hu et al. (2023) a combination of phenotypic drug sensitivity testing (pDST) and WGS was beneficial for the timely diagnosis and personalized treatment of bedaquiline-resistant TB. Similarly, as there is a lack of defined cutoffs and critical concentrations for conducting pDST of pretomanid, it is crucial to integrate conventional methods with WGS in determining its resistance (Bateson et al., 2022). In the past, the major limitation in using the WGS approach was the reliance on limited available mutation knowledge only. To overcome this limitation, WHO has developed a catalog of *Mtb* mutations and their association with phenotypic drug resistance to support personalized medicine in TB treatment. The catalog provides a reference standard for the interpretation of mutations conferring resistance to all first-line and a variety of second-line drugs (Walker et al., 2022b). Due to the complex bioinformatics analysis involved in processing WGS data, various non-commercial, freely available, and user-friendly software tools have been developed (such as TB Profiler, Mykrobe Predictor, TGS-TB, PhyResSE, and KvarQ). These software solutions enable medical personnel to rapidly diagnose TB, and interpret comprehensive drug resistance profiles directly from raw sequencing data (FASTQ files; Steiner et al., 2014; Feuerriegel et al., 2015; Sekizuka et al., 2015; Phelan et al., 2019).

The recurrence of TB is another factor that can complicate treatment individualization in TB patients (Dooley et al., 2011). Distinguishing the cause of TB recurrence is crucial to guide TB control and treatment. The potential of WGS lies in its ability to differentiate between relapse and reinfection, the two distinct mechanisms underlying TB recurrence (Nikolenka et al., 2021). WGS demonstrated its capability to differentiate between treatment failure (with the necessity to use a new drug regimen) and reinfection with a new strain in clinical trials evaluating novel anti-tuberculosis drugs (Gillespie et al., 2014). Another study utilizing WGS demonstrated a relatively high incidence of fluoroquinolone resistance during the second episode of TB relapse. These findings lead to caution when using fluoroquinolones for treating patients with recurrent TB and suggest the use of DST results for any treatment decisions (He et al., 2023).

To personalize TB treatment, it is crucial to consider if a mutation accurately identifies a strain with a higher minimum inhibitory concentration as well as if this mutation is linked to treatment failure (Chen et al., 2020). Recently, Domínguez et al. (2023) reviewed studies linking the treatment outcome with the presence of a specific mutation encoding resistance to first- and second-line anti-tuberculosis drugs. We consider these data to be very important, as they could prompt the clinician to consider a change in the treatment regimen in patients showing these mutations associated with resistance.

The utilization of WGS can also facilitate the identification and prediction of TB transmission. Recent research has demonstrated that the application of this technology enables the identification of transmission hotspots, both in countries with a low and high incidence of TB (Alaridah et al., 2019; Asare et al., 2020; Gordon et al., 2021; Dale et al., 2022). To prevent the spread of TB, it is crucial to describe the transmission chains in particular communities. Prisons are widely acknowledged to have an exceptionally high burden of TB (28 times greater) compared to the general population, serving as a reservoir for persistent MDR TB (Witbooi and Vyambwera, 2017; Anselmo et al., 2023). The recent findings demonstrated that 43 and 45.4% of TB cases among prisoners were due to direct transmission (Anselmo et al., 2023; Sanabria et al., 2023). WGS-based screening for TB before and after the transfer of prisoners could contribute to preventing TB transmission and reducing the number of TB cases. Migrants are another at-risk demographic group, accounting for up to 40–60% of TB cases in many high-income countries (Iñigo et al., 2013; Ospina et al., 2016). WGS-based cross-border surveillance is essential to present TB epidemiological monitoring to differentiate between imported and recent transmission cases (Abascal et al., 2019). Overall, TB tracing with WGS may be an effective strategy in the treatment and/or chemoprophylaxis of close contacts.

One of the limitations of WGS is its reliance on obtaining high-quality genomic DNA from cultured *Mtb* isolates. The cultivation process can take several weeks, presenting an additional disadvantage of this technology from a clinical perspective (Dookie et al., 2022; Walker et al., 2022a). Increasing interest is focused on culture-free target-based NGS (Cabibbe et al., 2020). The use of direct sputum samples for analysis makes targeted NGS an attractive method, primarily because of its capability to provide results more rapidly (Mansoor et al., 2023). Recently, several studies have demonstrated the efficacy of culture-free targeted NGS for the detection of drug-resistant *Mtb* using Deeplex Myc-TB (Genoscreen, Lille, France). This assay achieved excellent sensitivity and specificity in the detection of resistance to 13 anti-tuberculosis drugs compared to pDST and could be a breakthrough in the rapid diagnosis of MDR TB in routine practice (Bonnet et al., 2021; Feuerriegel et al., 2021; Jouet et al., 2021). Another benefit of this assay is its ability to be utilized in conjunction with nanopore-based DNA sequencing instruments, such as the MinION (Oxford Nanopore Technologies (ONT), Oxford, UK). This characteristic makes it especially advantageous in settings with limited resources (Cabibbe et al., 2020). These sequencing instruments exhibit portability, resilience, and cost-effectiveness, which renders them suitable for potential use in point-of-care settings to perform targeted NGS (Dippenaar et al., 2022; Hall et al., 2023). This capability has the potential to revolutionize TB DST and personalize the treatment process. Moreover, Sibandze et al. conducted focused NGS to detect drug resistance directly from stool samples provided by individuals with TB. This offers a valuable opportunity to gather essential diagnostic information for TB patients who encounter challenges in providing respiratory specimens (Sibandze et al., 2022).

The choice of method depends on the specific research or diagnostic goals, as well as the available resources and expertise. The field of NGS is continually evolving, and new methods and technologies are being developed to improve our understanding and management of TB.

## 5. Conclusion

PPPM can help improve TB prevention, diagnosis, and treatment by considering individual differences in risk and response to interventions. Adopting this approach can also help engage patients and communities in managing TB, leading to better health outcomes and reduced disease burden. Currently, the most promising strategies in PPPM for TB include the use of AI, genetic screening, and NGS.

More specifically, AI has the potential to assist in the prediction and diagnosis of TB in developing countries where advanced radiological expertise is lacking. Additionally, this technology may be useful in predicting the effectiveness of treatment regimens and acute adverse effects during therapy and tracking TB cases. Genetic screening can also have a crucial function in anticipating active TB and ensuring the efficacy of therapy.

Recently, a variety of mutations in genes related to immune function, CYP450, and certain miRNAs linked to LTBI reactivation have been identified. Furthermore, identifying mutations in certain genes can predict the likelihood of adverse reactions and the efficacy of treatment. Based on these assumptions, it may be worth considering the clinical relevance of genetic screening and its potential application in routine diagnostics.

The development of novel molecular diagnostic methods has also made a huge contribution to the personalization of therapy and the prevention of TB transmission. Utilizing the full potential of NGS, comprehensive insights into the phylogenetic lineage of infecting strains can provide clinicians with valuable information regarding the likelihood of the strain developing additional drug resistance. These innovative approaches in TB treatment signify a new era in the management of MDR-TB that will aid in mitigating treatment failure and ongoing transmission.

In contrast to the aforementioned advantages, there are several potential knowledge gaps and areas for future research regarding the management of TB and the roles of AI, genetic screening, and WGS. The successful integration of AI into clinical practice for TB management requires a holistic approach that addresses technical, regulatory, educational, and usability aspects. Research in these areas can pave the way for more effective and widespread use of AI to combat TB and improve patient outcomes. In the realm of genetic screening for TB management, research should focus on understanding population-specific variations, and assessing the cost-effectiveness of these screening methods. It’s essential to determine the accuracy of genetic markers, integrate them effectively into clinical decision-making, and provide ethical patient counseling. Additionally, research should explore the impact on healthcare systems and potential contributions to drug development. Global collaboration and data sharing are also vital for advancing this field. In summary, addressing these knowledge gaps and conducting research in these areas can contribute to more effective TB control and management strategies, ultimately reducing the global burden of this disease.

## Author contributions

MD wrote the manuscript. IS and IP edited the manuscript. JM supervised and finalized the manuscript. All authors contributed to the study’s conception and design, read, and approved the final manuscript.
